# Decision making in young people at familial risk of depression

**DOI:** 10.1017/S0033291714001482

**Published:** 2014-06-23

**Authors:** Z. N. Mannie, C. Williams, M. Browning, P. J. Cowen

**Affiliations:** University Department of Psychiatry, Warneford Hospital, Oxford, UK

**Keywords:** At risk, decision making, depression, reward, risky choice

## Abstract

**Background:**

Major depression is associated with abnormalities in reward processing at neural and behavioural levels. Neural abnormalities in reward have been described in young people at familial risk of depression but behavioural changes in reward-based decision making have been less studied in this group.

**Method:**

We studied 63 young people (mean age 18.9 years) with a parent with a diagnosis of major depression but who had never been depressed themselves, that is with a positive family history of depression (the FH+ group). Participants performed the Cambridge Gambling Task (CGT), which provides several measures of decision making including deliberation time, quality of decision making, risk taking, risk adjustment and delay aversion. A control group of 49 age- and gender-matched young people with no history of mood disorder in a first-degree relative undertook the same task.

**Results:**

Both FH+ participants and controls had low and equivalent scores on anxiety and depression self-rating scales. Compared to controls, the FH+ participants showed overall lower risk taking, although like controls they made more risky choices as the odds of a favourable outcome increased. No other measures of decision making differed between the two groups.

**Conclusions:**

Young people at increased familial risk of depression have altered risk taking that is not accounted for by current affective symptomatology. Lowered risk taking might represent an impairment in reward seeking, which is one of several changes in reward-based behaviours seen in acutely depressed patients; however, our findings suggest that decreased reward seeking could be part of a risk endophenotype for depression.

## Introduction

Loss of interest and pleasure (anhedonia) are cardinal symptoms of acute major depression and are generally regarded as representing abnormalities in reward mechanisms (Eshel & Roiser, 2010). Although anhedonic symptoms usually remit as depression improves, it has been suggested that the neurobiological mechanisms underlying anhedonia could represent an endophenotype of depression that might manifest in behavioural and neural changes outside acute depressive episodes (Hasler *et al*. 2004). In agreement with this proposal, we have shown that recovered depressed patients withdrawn from medication continue to manifest abnormal neural responses in the ventral striatum and orbitofrontal cortex to food reward (McCabe *et al.* 2009).

More recently, we found that that young people with no personal history of depression but at increased familial risk also have impaired neural processing of reward, particularly affecting the orbitofrontal cortex and anterior cingulate cortex (McCabe *et al.* 2012). These cortical areas are known to be involved in reward-based learning (Murray *et al.* 2011) but our imaging study was not designed to explore differences in reward-based behaviours.

Studies in adult patients with major depression have shown impaired decision making on reward-based tasks, notably in terms of a ‘conservative’ response strategy, where depressed patients fail to take a risk even when the odds of a favourable outcome are high (Murphy *et al.* 2001). Forbes *et al.* (2007) obtained a similar finding in boys with depression aged 10–11 years who, under conditions of high reward probability, failed to prefer a high magnitude reward over a low one. Moreover, in non-depressed participants this pattern of response predicted future depression. This observation is consistent with a recent study using the Cambridge Gambling Task (CGT), in which in a group of young people at familial risk of depression, those with current depressive symptoms and those who developed depression subsequently also showed decreased risk taking compared to those who remained well over follow-up (Rawal *et al*. 2013). However, the latter study did not include a normal control group for comparison.

The purpose of the present study was to assess reward-based decision making in young people at increased familial risk of depression in comparison to a control group not at risk. We also used the CGT, which provides several measures of reward-based decision making including risky choice under different reward contingencies.

## Method

### Participants and psychosocial measures

We recruited 63 young people (39 women, 24 men) with a mean age of 18.9 ± 1.0 years (range 16–20 years) who had never personally suffered from depression but who had a biological parent with a history of major depression (FH+). Potential participants were assessed with the SCID-I to exclude a personal current or previous history of major depression (First *et al.* 1997). The presence of major depression in a parent was assessed by the family history method using the participant as an informant (Andreasen *et al.* 1986). The criteria used included description of the symptoms of major depression together with the prescription of specific antidepressant treatment, either psychotherapy or medication. This was followed up by direct verification from the affected parent (either by telephone or in writing); where parental history could not be verified, participants were excluded. A history of bipolar disorder in a parent was an exclusion criterion. We also recruited 49 controls (30 women and 19 men) with a mean age of 19.1 ± 0.8 years (range 16–20 years) who were determined by the same instruments to have no current or past history of major depression and no history of depression in a biological parent or other first-degree relative. The two groups were matched for age and gender. All participants were in full-time education with all but six being students at Oxford University or Oxford Brookes University. The remaining participants were in secondary school.

At baseline, participants were assessed for current mood and anxiety symptoms with the Hospital Anxiety and Depression Scale (HADS; Zigmond & Snaith, 1983) and the State–Trait Anxiety Inventory (STAI; Spielberger *et al.* 1983), and the Perceived Stress Scale (PSS; Cohen *et al.* 1983) was used to obtain a measure of subjective stress over the past month. Adverse life events and the impact of these events on emotional well-being were assessed with the Life Events Rating Scale (LERS), which assesses adverse events at two time points: first, at a distal time point that includes childhood adversity, and second, events experienced in the past year (Goodyer *et al.* 1997). We assessed personality with the Eysenck Personality Questionnaire (EPQ; Eysenck & Eysenck, 1975). Quality of perceived parenting style for the first 16 years of life was assessed with the Parental Bonding Instrument (PBI), obtaining both maternal and paternal PBI scores (Parker, 1979). IQ was assessed with the National Adult Reading Test (NART). All participants gave full informed consent to the study, which was approved by the local ethics committee.

### The CGT

The CGT (Cambridge Neuropsychological Test Automated Battery, CANTAB; www.camcog.com; Rogers *et al.* 1999; Clark *et al.* 2008) assesses decision making and risk-taking behaviour outside a learning context. Participants are shown 10 red and blue boxes at the top of the screen and the ratio of red to blue boxes varies between 9:1, 8:2, 7:3, 6:4 and 5:5, and *vice versa* in a pseudo-random order. Participants are informed that a yellow token is hidden inside one of the boxes and are asked to indicate in which colour box is the token most likely to be hidden, by pressing the colour (RED or BLUE) in a response panel at the bottom of the screen. Following their response, the participants indicate confidence in their selection by betting a proportion of points they are allocated (starting from 100 points). Besides confidence in selection, this measure also assesses the willingness to risk the points they already possess or have accumulated for further real or perceived reward. On each trial five bets are offered, and each bet represents a fixed percentage of the current total points score (5, 25, 50, 75 and 95%). Possible bets are presented sequentially in a box on the right of the display and participants touch the box to select the bet. If correct, the bet value is added to their total points on the left of the panel, and if incorrect, it is subtracted from the total points. Participants are asked to accumulate as many points as possible. Following the response, the location of the token is revealed. Participants perform the task in four blocks of two separate conditions (ascending and descending bet value) and the condition order was counterbalanced across participants. In the ascending condition, bets increased at 2.5-s time intervals from 5% to 95% until participants made their selection. This means that if participants bet at the first value presented, they bet only 5% of their total points, and if they wait for the highest value they bet 95% of their total points. In the descending condition bets start from 95% and decrease to 5%. Low bets in the ascend condition and high bets in the descend condition reveal an impulsive betting strategy, whereas high bets in both conditions reveal a risk-taking strategy.

From the first stage of the task (indicating the likely colour of the box in which the token is hidden), the outcome measures are deliberation time and quality of decision making. Deliberation time is the mean latency from presentation of coloured boxes to participant selection. Quality of decision making refers to the proportion of trials in which the more likely outcome is chosen. From the gamble stage, the outcome measures are risk taking, risk adjustment and delay aversion. Risk taking refers to the mean proportion of current points that the subject stakes on each gamble when the more likely outcome is selected, and can be regarded as an index of reward seeking or loss aversion. Risk adjustment measures the degree to which a subject varies their risk taking in response to the ratio of red to blue boxes on each trial. Delay aversion is the difference between the risk-taking score in the descend and ascend conditions.

### Statistics

All data were analysed with SPSS version 20 (SPSS Inc., USA). Analyses were conducted using unpaired *t* tests (two-tailed) or an ANOVA, with ‘group’ (FH+ participants *versus* controls) as a between-subjects factor. Where necessary, covariates were added. Categorical data were analysed with the *χ*^2^ test and correlations were carried out using Pearson's product moment.

### Ethical standards

All procedures contributing to this work comply with the ethical standards of the relevant national and institutional committees on human experimentation and with the Helsinki Declaration of 1975, as revised in 2008.

## Results

There were no group differences in baseline measures of current mood and anxiety states, trait anxiety, perceived stress, life events and neuroticism (all *p* values > 0.15). There were group differences in IQ (see Table 1), which was subsequently used as a covariate. CGT performance did not differ significantly between FH+ and control participants for the following outcomes: quality of decision making (*F*_1,110_ = 0.41, *p* = 0.53), deliberation time (*F*_1,110_ = 1.00, *p* = 0.32), delay aversion (*F*_1,110_ = 0.47, *p* = 0.50) and risk adjustment (*F*_1,110_ = 0.003, *p* = 0.96) (Table 2). However, there was a significant group difference in risk taking (*F*_1,110_ = 6.52. *p* = 0.012), with the FH+ participants taking fewer risks irrespective of how high or low the probability was of a favourable outcome (Table 2). This finding remained significant when an ANCOVA was performed with IQ as a covariate (*F*_2,109_ = 5.51, *p* = 0.02).

**Table 1.** Participant characteristics

	FH+ (*n* = 63)	Controls (*n* = 49)	*p* value
Age (years)	18.9 (1.1)	19.1 (0.8)	0.3
Gender (M/F)	24/39	19/30	0.9
NART IQ	119.7 (4.3)	117.3 (4.7)	0.006
HADS-D	1.6 (2.4)	1.5 (1.7)	0.4
HADS-A	4.3 (3.0)	4.1 (3.0)	0.8
STAI state	33.0 (8.8)	32.4 (8.3)	0.7
STAI trait	34.2 (9.5)	34.2 (8.3)	1.0
PSS	13.0 (5.7)	13.4 (5.7)	0.7
LERS recent	0.9 (1.1)	0.7 (1.0)	0.5
LERS lifetime	0.5 (0.6)	0.4 (0.7)	0.5
PBI maternal care	30.5 (0.8)	30.7 (0.9)	0.8
PBI overprotection	10.2 (0.9)	10.1 (1,0)	1.0
PBI paternal care	27.1 (0.8)	27.2 (1.0)	1.0
PBI overprotection	7.5 (0.8)	7.3 (0.9)	0.9
EPQ-N	8.5 (6.2)	6.9 (4.8)	0.1

FH+, Participants with a positive family history of depression; M, male; F, female; NART, National Adult Reading Test; HADS, Hospital Anxiety and Depression Scale (D, depression subscale; A, anxiety subscale); STAI, State–Trait Anxiety Inventory; PSS, Perceived Stress Scale; LERS, Life Events Rating Scale; PBI, Parental Bonding Instrument; EPQ-N, Eysenck Personality Questionnaire – Neuroticism.

Data are presented as mean (standard deviation).

**Table 2.** Results on the Cambridge Gambling Task (CGT)

	FH+ (*n* = 63)	Controls (*n* = 49)	*p* value
Delay aversion (%)	18 (2.1)	16 (1.8)	0.49
Deliberation time (ms)	1612 (43)	1692 (73)	0.32
Quality decision (%)	99 (0.5)	99 (0.4)	0.53
Risk adjustment	1.8 (0.10)	1.8 (0.10)	0.96
Risk taking/seeking (%)	60 (1.4)	65 (1.3)	0.012

FH+, Participants with a positive family history of depression.

Data are presented as mean (standard error of the mean).

**Fig. 1.**
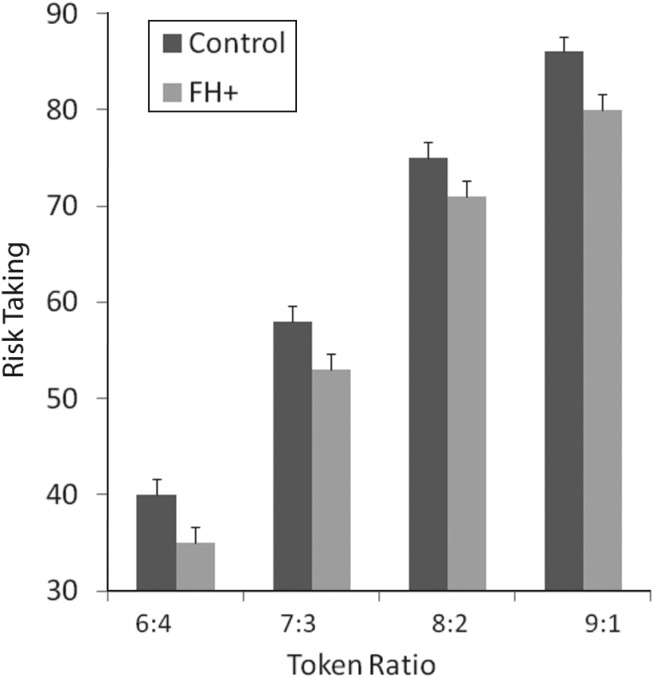
Risk taking shown as mean (standard error of the mean) percentage of total points bet when a more likely outcome is selected, at differing ratios (likelihood) of selections being correct. The group with a positive family history of depression (FH+) bet significantly less, as shown by the main effect of group on ANOVA (*F*_1,110_ = 6.42, *p* = 0.013).

Analysis of the proportion of points bet as a function of participant group and the odds of winning revealed main effects of both group (*F*_1,110_ = 6.42, *p* = 0.013) and odds ratio (*F*_3,110_ = 562.7, *p* = 0.000). There was no interaction between group and odds ratio. As can be seen from Fig. 1, these results arise because participants in the FH+ group wagered fewer points than the control group across all of the different bets. In other words, although FH+ participants were generally less risk seeking than the control group, they used information about the probable outcome of each decision to modulate their bet in the same way as the control group.

When gender was added as a covariate, there were main effects of gender on deliberation time (*F*_1,110_ = 4.20, *p* = 0.043) and risk adjustment (*F*_1,110_ = 5.92, *p* = 0.017), where women had longer deliberation times than men (1708 *v.* 1548 ms, *p* = 0.05) and less risk adjustment as the odds of winning increased (1.7 *v.* 2.0, *p* = 0.017). There were no other significant main effects of gender or group by gender interactions (all *p* values > 0.05).

No significant correlation was found between symptom scores of depression and anxiety and overall proportion bet or risk taking (all *p* values > 0.05) in all the participants taken together or in the FH+ participants considered alone. There was no significant correlation between IQ and risk taking (*r* = –0.079, *p* = 0.40) or between IQ and any of the other indices of the CGT.

## Discussion

We assessed reward-based decision making in young people at increased risk of depression based on having a depressed parent. Compared to the control group, we found no differences in performance in terms of quality of decision making, deliberation time and degree of impulsivity. However, FH+ participants generally took fewer risks in their betting irrespective of the probability of a favourable outcome, although they did show the expected increase in the proportion staked as the odds of success increased (risk adjustment). Thus, they seemed to demonstrate a generally lower level of risk taking, that is a conservative response style.

Our findings have interesting similarities to another study using the CGT in young people (aged 10–18 years) who had a parent with recurrent depression. In these participants, both current depressive symptoms and future risk of depression were associated with a decrease in risk taking, similar to that demonstrated by the FH+ participants in the current study (Rawal *et al.* 2013). We are currently following up our own participants to determine whether a similar finding is obtained, as many of them have not yet passed though the period of elevated risk of depression (Beardslee *et al.* 1998).

Using the CGT, Murphy *et al.* (2001) found that acutely depressed patients bet less than controls on favourable outcomes, thus also showing a conservative response style. However, in this study depressed patients also increased their bets at a slower rate than controls, as the odds of a favourable outcome increased. Both the latter studies showed effects of current depressive symptoms on responses in the CGT. However, in our study we found no correlations between risk taking and symptomatic scores on the HADS, although generally ratings of anxiety and depression in participants were low.

Forbes & Dahl (2012) summarized studies that used different tasks to examine reward-based decision making in adult and adolescent patients with established depressive disorders. Although a variety of abnormalities were reported, there was a pattern for depressed people to make more conservative choices when seeking rewards, to demonstrate less reward response bias and to be less sensitive to changing reward contingencies (Corwin *et al.* 1990; Forbes *et al*. 2007; Pizzagalli *et al.* 2008). Vrieze *et al.* (2013) recently reported that reduced reward learning in depressed patients was associated with a decreased likelihood of responding to treatment at 8 weeks. Adults with depression can also exhibit impaired ‘delay discounting’, that is the ability to defer response to obtain a larger, albeit delayed, reward or to shift strategy to improve decision making (Must *et al.* 2013). However, in our study, impaired delay discounting did not seem to be a feature of the performance of FH+ participants.

Several possible psychological mechanisms could underlie the diminished risk taking seen in FH+ participants (Treadway & Zald, 2011). For example, FH+ participants may be hyposensitive to rewards, that is they may value rewards less, or be oversensitive to loss (punishment) and therefore be risk averse; both these abnormalities have been described in depressed patients and could be associated with the conservative approach to risk seen in the present study (Eshel & Roiser, 2010). In addition, FH+ participants may have a lower overall assessment of the likelihood of a positive outcome, which lowers the potential utility of a risky choice (Rangel *et al.* 2008).

In a recent neuroimaging study of taste reward and punishment, we found that, relative to controls, FH+ participants showed diminished responses to chocolate reward in the orbitofrontal cortex whereas aversive taste stimuli produced increased activations in the lateral orbitofrontal cortex (McCabe *et al.* 2012). Orbitofrontal regions are thought to be involved in updating neural representations of objects to represent their current biological value (Murray *et al.* 2011; Rushworth *et al.* 2011). Therefore, these neural changes are consistent with the notion that, relative to controls, values of rewards and punishments are negatively shifted in young people at risk of depression. In the same imaging study, we found that FH+ participants showed blunted neural responses to both rewarding and aversive stimuli in the anterior cingulate cortex, a brain region involved in linking actions to negative and positive outcomes (Glascher *et al.* 2009; Murray *et al*. 2011). Hence, impaired activity in the anterior cingulate cortex could underpin difficulties in the use of rewarding and aversive information to guide risky decision making and perhaps lead to a more conservative risk-taking strategy. In this respect it is of interest that we also found blunted neural responses in the anterior cingulate cortex in FH+ participants undertaking an emotional Stroop task; this suggests that familial risk of depression could be associated with a more general difficulty in integrating cognitive and emotional information in this brain region (Mannie *et al.* 2008).

Having a parent with a history of recurrent major depression is an important risk factor for the development of depression. Indeed, it has been estimated that, by young adulthood, around 40% of people with a biological parent affected by depression will have developed depression themselves (Beardslee *et al.* 1998). Depression is a multifactorial disorder and it is not possible in our study to identify how far genetic or environmental risk factors play a predominant role in the manifestation of altered reward seeking. Nevertheless, the psychosocial measures we obtained failed to show significant differences between FH+ participants and controls in several important respects, including relationships with parents, recent and remote life events and current levels of stress. In addition, current levels of anxiety and depression were found to be very similar in FH+ participants and controls. A small but significant difference in IQ did not seem to be responsible for the difference in risk taking between the two participant groups, although it must be acknowledged that the NART is only an approximate measure of intelligence. In general, studies of risky decision making have not found reliable gender differences in responses; however, the lower risk adjustment seen in women in the present study replicates a previous report with the CGT (Deakin *et al.* 2004).

In conclusion, our study suggests that young people at increased familial risk of depression have altered risk taking that is not accounted for by current affective symptomatology. The conservative risk-taking strategy that we have identified is not necessarily maladaptive and might even be beneficial in some circumstances, for example a particularly unpredictable environment. However, the fact that this kind of risk strategy is seemingly associated with the future risk of depression (Forbes *et al.* 2007; Rawal *et al.* 2013) suggests that it may have adverse consequences, although a causal link between conservative risk strategy and subsequent depression has not been conclusively established.

A possible mechanism that might explain decreased risk taking is impaired reward seeking. This is one of several changes in reward-based behaviours seen in acutely depressed patients but our findings suggest that decreased reward seeking could be part of a risk endophenotype for depression. A tendency to withdraw from risky choices even when a positive outcome is likely may have a subtle but negative impact on productivity and social interactions. This ties in with the notion that part of the way that genetic risk of depression might be expressed is through the inadvertent ‘selection’ of suboptimal interpersonal and occupational environments (Kendler & Karkowski-Shuman, 1997).
